# ‘How long do you think?’ Unresponsive dying patients in a specialist palliative care service: A consecutive cohort study

**DOI:** 10.1177/02692163241238903

**Published:** 2024-04-23

**Authors:** Tricia O’Connor, Wai-Man Liu, Juliane Samara, Joanne Lewis, Catherine Paterson

**Affiliations:** 1 Clare Holland House, Canberra Health Services, North Canberra Hospital, Canberra, ACT, Australia; 2 Caring Futures Institute, Flinders University, Bedford Park, SA, Australia; 3 Research School of Finance, Actuarial Studies and Statistics, Australian National University, Canberra, ACT, Australia; 4 School of Nursing and Health, Avondale University, Wahroonga, NSW, Australia; 5 Central Adelaide Local Health Network, Adelaide, SA, Australia; 6 School of Nursing, Midwifery and Public Health, University of Canberra, Bruce, ACT, Australia; 7 Robert Gordon University, Aberdeen, Scotland, UK

**Keywords:** Cohort study, palliative care, terminally ill, prognosis, Australia-modified Karnofsky Performance Status, terminal care, unresponsive, dying, prognostication

## Abstract

**Background:**

Predicting length of time to death once the person is unresponsive and deemed to be dying remains uncertain. Knowing approximately how many hours or days dying loved ones have left is crucial for families and clinicians to guide decision-making and plan end-of-life care.

**Aim:**

To determine the length of time between becoming unresponsive and death, and whether age, gender, diagnosis or location-of-care predicted length of time to death.

**Design:**

Retrospective cohort study. Time from allocation of an Australia-modified Karnofsky Performance Status (AKPS) 10 to death was analysed using descriptive narrative. Interval-censored survival analysis was used to determine the duration of patient’s final phase of life, taking into account variation across age, gender, diagnosis and location of death.

**Setting/participants:**

A total of 786 patients, 18 years of age or over, who received specialist palliative care: as hospice in-patients, in the community and in aged care homes, between January 1st and October 31st, 2022.

**Results:**

The time to death after a change to AKPS 10 is 2 days (*n* = 382; mean = 2.1; median = 1). Having adjusted for age, cancer, gender, the standard deviation of AKPS for the 7-day period prior to death, the likelihood of death within 2 days is 47%, with 84% of patients dying within 4 days.

**Conclusion:**

This study provides valuable new knowledge to support clinicians’ confidence when responding to the ‘how long’ question and can inform decision-making at end-of-life. Further research using the AKPS could provide greater certainty for answering ‘how long’ questions across the illness trajectory.

**What is already known about the topic?**

- A decrease in conscious state is an accepted sign of imminent death.
- Planning and preparing for death improves quality of dying and death outcomes.
- Lack of awareness of approaching death is associated with negative bereavement outcomes.

**What this paper adds?**

- Findings identify an association between the Australia-modified Karnofsky Performance Status 10 score and timeframes to death.
- The length of time to death for most patients once they are comatose or barely rousable is 2 days.
- However, this study highlights the importance of clinicians acknowledging and conveying uncertainty in prognostic accuracy.

**Implications for practice, theory or policy**

- The recognition of imminent death needs to be communicated clearly, with findings from this research used to support and inform practice and policy on end-of-life care.
- Care can be targeted to support good patient and family outcomes in the last days of life.
- The research findings support communication and decision-making and can improve end-of-life clinical care planning.

## Introduction

Predicting the time of death is fraught with significant uncertainty, and a subject many healthcare professionals try to avoid.^1–3^ ‘How long do you think?’ is a question that is asked regularly of healthcare professionals, and predicting death continues to be a challenge and source of distress for the patient, family and healthcare professionals.^4^ Research indicates that the closer to death the patient is, the higher the likelihood of a more accurate prediction.^5–7^ Commonly however when healthcare professionals do predict, even specialist palliative care clinicians frequently overestimate and predict that patients will live longer, even in the last days of life.^5,7,8^

When the patient is unresponsive and clearly dying, the family often wish to know how long before their loved one’s death. A decrease in conscious state (where the patient becomes unresponsive), has been accepted as a sign of imminent death, together with resulting swallowing difficulty and reduced oral intake.^9–11^ Despite much research, such as clinician prediction of survival,^12–14^ continuous monitoring and observation of signs and symptoms of imminent death^7,9,15–17^ and the use of prognostic scales,^6,8,9^ significant uncertainty in accurate prognostication remains in practice.

The informed knowledge of how many hours or days a patient has left is vital for families and clinicians to prepare, plan and guide decision-making for care.^5,7,8,18,19^ Previous research provides broad estimations of time to death for dying patients of between 1 and 3 days.^9,20–24^ Other research compared survival time between cancer and non-cancer diagnosis,^21,25^ including covariates such as gender^23,24,26^ and age.^20,27^ Researchers however acknowledge the lack of accuracy,^5^ level of uncertainty and unreliability of prognostic tools^28^ and the risk of overestimation^7,8^ when clinicians predict time till death. Despite this work, none of these studies focussed solely on the unresponsive dying person to determine the time till death; and as such cannot be used to answer the ‘how long’ question at end-of-life with confidence.

Interrogating this gap in knowledge could improve prognostication significantly, with a view to guiding decision-making for clinicians and families in the last days and hours of life. The aim of this research was to determine how long dying patients in a palliative context are unresponsive prior to death. The secondary aim was to determine whether age, gender, primary diagnosis or setting of care predicted length of time to death.

## Methods

This was a large retrospective cohort study.

### Setting

The context for this research is a specialist palliative care service in regional Australia, which includes a 19-bed in-patient hospice unit and associated community services (home-based palliative care). A specialist palliative aged care team provide in-reach specialist palliative care services to residents living across 28 aged care facilities, working in partnership with the staff of these facilities. Other arms of the service, such as outpatients, motor neurone disease clinics and hospital consultations, were not included in the study as these services do not regularly document Australia-modified Karnofsky Performance Status (AKPS) scores.^29^

### Participants

The study population were patients who received specialist palliative care. Inclusion and exclusion criteria:

*Inclusion criteria*
i. patients who were 18 years or over; and
ii. whose last episode of care ended with death between 1 January 2022 and 31 October 2022, and
iii. had more than one recorded AKPS score, with scores documented for longer than 24-h, and
iv. were cared for as in-patients in a 19-bed hospice; or
v. were cared for in the community by a community specialist palliative care team; or
vi. were cared for in an aged care home by the visiting specialist palliative care team.
*Exclusion criteria*
i. patients cared for by other arms of the specialist palliative care service

### Data collection: Tool

The Australia-modified Karnofsky Performance Status (AKPS) is a reliable and validated tool which measures performance status.^29^ An overall performance score of 100% represents full physical function, with decreasing increments of 10 indicating a reduced self-care ability. An AKPS score of 20 indicates the patient is bedfast and requires extensive nursing care, an AKPS 10 is scored to represent a patient who is ‘comatose or barely rousable’,^29^ and 0% when the patient has died. Based on the AKPS criteria unresponsive dying patients score an AKPS 10, that is, they are comatose or barely rousable. AKPS data is routinely recorded and collated by the internationally recognised Palliative Care Outcomes Collaboration, inclusive of 177 services that provide palliative care across Australia.^30^ The data for this project was gathered from a palliative care service which was part of the Palliative Care Outcomes Collaboration. AKPS scores provide a common clinical language for palliative care,^31^ defining and identifying imminently dying patients.

AKPS assessments were completed on average twice a day for hospice in-patients, and at each clinical encounter for community-based and aged care home patient encounters. AKPS scores were prospectively documented from the time of admission to the service, and all scores were accounted for during retrospective analysis from the date of death.

### Data collection: Method

To meet the objectives of this retrospective cohort study data was collected from PalCare, a web-based palliative care patient information management system (http://www.palcare.com.au/). PalCare were contracted to provide a tailored report of existing data from the local PalCare database to answer the research questions. Further information from individual patient records was manually retrieved to clarify relevant details, such as any missing demographics or diagnoses that had not been entered into the correct fields.

Data were extracted to an Excel spread sheet and included the following variables: date of birth, gender, ethnicity, Aboriginal and Torres Strait Islander status, date and site of death, specialist palliative care service type, postcode, cancer or non-cancer and diagnostic cohorts: solid organ failure, neurological conditions, cardiovascular disease, Alzheimer’s disease/dementia, others and unknown/undefined. Only the recorded primary diagnosis was included, regardless of comorbidities. All AKPS scores and dates of assessments from admission until death were extracted.

### Analysis

Data was quality checked by all members of the research team to ensure it was accurate prior to analysis. Data was verified with clinical records by research clinicians to determine the clinical context of AKPS scores and explain any outliers or anomalies, and to improve the integrity of the data. Descriptive statistics were used to summarise the demographics and characteristics of the cohort. For multi-group comparison (location), ANOVA was used. Since there is no right-censoring in the data, Kruskal-Wallis test was used to compare days-to-death from AKPS 10 across the different groups (cancer, primary diagnosis, age and location). Interval-Censored Cox Proportional Hazards model^32,33^ was used to analyse the left censored data, that is, patients may have already been comatose or barely rousable for a time before assessment. All data management and analyses were executed in STATA 16 statistical software (StataCorp LP, College Station, TX). For all statistical tests, a *p*-value less than 0.05 was considered statistically significant.

### Ethical approval

Ethics approval was obtained through Calvary Healthcare Human Research Ethics Committee (10-2022, approval date 18-08-2022). Cross-institutional approval from the University of Canberra and Australian National University Ethics Committees was also obtained. Individual patient consent was not required as data collected was routine clinical data that was de-identified and aggregated.

## Results

**Figure 1.**
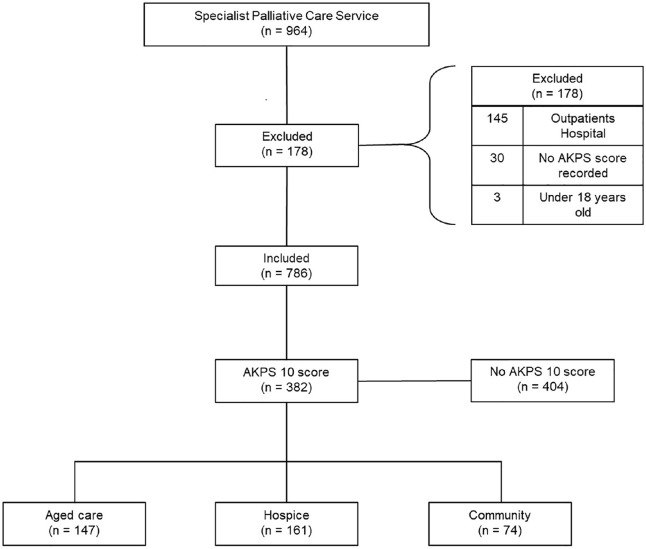
STROBE flow diagram: Patient inclusion

A total of 964 patients cared for by the specialist palliative care service died within the study period (see Figure 1). Collectively there were 8930 AKPS data points recorded across the 786 included patients. Of included patients, a total of 382 patients (49%) had an AKPS score of 10 recorded prior to death. Fifty seven percent of patients were over the age of 80 years (*n* = 219). Only 0.8% (*n* = 3) were documented as identifying as Aboriginal and Torres Strait Islander (see Table 1). Of those who had an AKPS of 10 and were aged between 18 and 60 years, 91% (*n* = 20) had a cancer diagnosis. The corresponding percentage for age groups between 61 and 80 years of age, and over 80 years were 74% (*n* = 104) and 32% (*n* = 69), respectively.

**Table 1.** Demographics.

	Full sample		Patients in AKPS 10	
	Mean/*N*	%	Mean/*N*	%
Total number of patients	786	–	382	–
Aged care	372	47	147	39
Hospice	252	32	161	42
Community	162	21	74	19
Age (Mean)	81.3	–	80.8	–
⩽60	45	6	22	6
61–80	279	35	141	37
>80	462	59	219	57
Gender				
Male	354	45	174	46
Female	432	55	208	54
Primary diagnosis				
Cancers	355	45	193	51
Solid organ failure	72	9	28	7
Neurological conditions	68	9	31	8
Cardiovascular disease	86	11	39	10
Alzheimer’s disease/dementia	160	20	70	18
Others	45	6	21	6
Unknown/undefined	2	0	0	0

To specifically answer the ‘how long do you think . . .’ question, the mean, median and standard deviation across the three areas of care were examined. The average number of days to death after change to AKPS 10 was 2.1 and the corresponding median value is 1 (49% of patients were comatose or barely rousable for greater than or equal to 1 day; Table 2).

**Table 2.** Time to death after change to AKPS 10 (*N* = 382 patients).

Panel A: Overall										
	Time to death (days)									
	Mean			Median				*SD*			
After first change to										
AKPS 10		2.1			1				2.3		
Panel B: Across locations: Aged Care, Hospice and Community.										
	Time to death (days)									ANOVA *p*-value
	Aged care			Hospice			Community			
Mean	Median	SD	Mean	Median	SD	Mean	Median	SD		
After first change to AKPS 10	2.1	2	1.9	2.0	2	1.7	2.2	1	2.9	0.68

There were seven outliers (1.8%) who lived longer than a week, where six survived between eight and 11 days and one for 18 days. Six of these patients were older than 70 years, with three cared for in aged care homes, three in the community and one in the hospice. One patient, who was under 40 years of age with a malignant neurological disorder, lived for 18 days with a consistent AKPS score of 10.

Once patients were allocated an AKPS score of 10 there were minimal changes to the score (ie returning to AKPS 20 or above). No statistically significant difference was found in the time to death across locations (Table 2) or the number of non-zero AKPS changes (Table 3) after a change to AKPS 10.

**Table 3.** Non-zero AKPS Score change after first scored at AKPS 10 (*N* = 124 patients).

Panel A: Overall										
	Total number of non-zero change									
	Mean			Median			SD			
After first change to AKPS 10		1.3				1				0.7		
Panel B: Across three locations: aged care, hospice and community.										
	Total number of non-zero change									ANOVA *p*-value
	Aged care (*N* = 12)			Hospice (*N* = 94)			Community (*N* = 23)			
Mean	Median	SD	Mean	Median	SD	Mean	Median	SD		
After first change to AKPS 10	1.1	1	0.3	1.2	1	0.6	1.5	1	0.9	0.22

**Figure 2.**
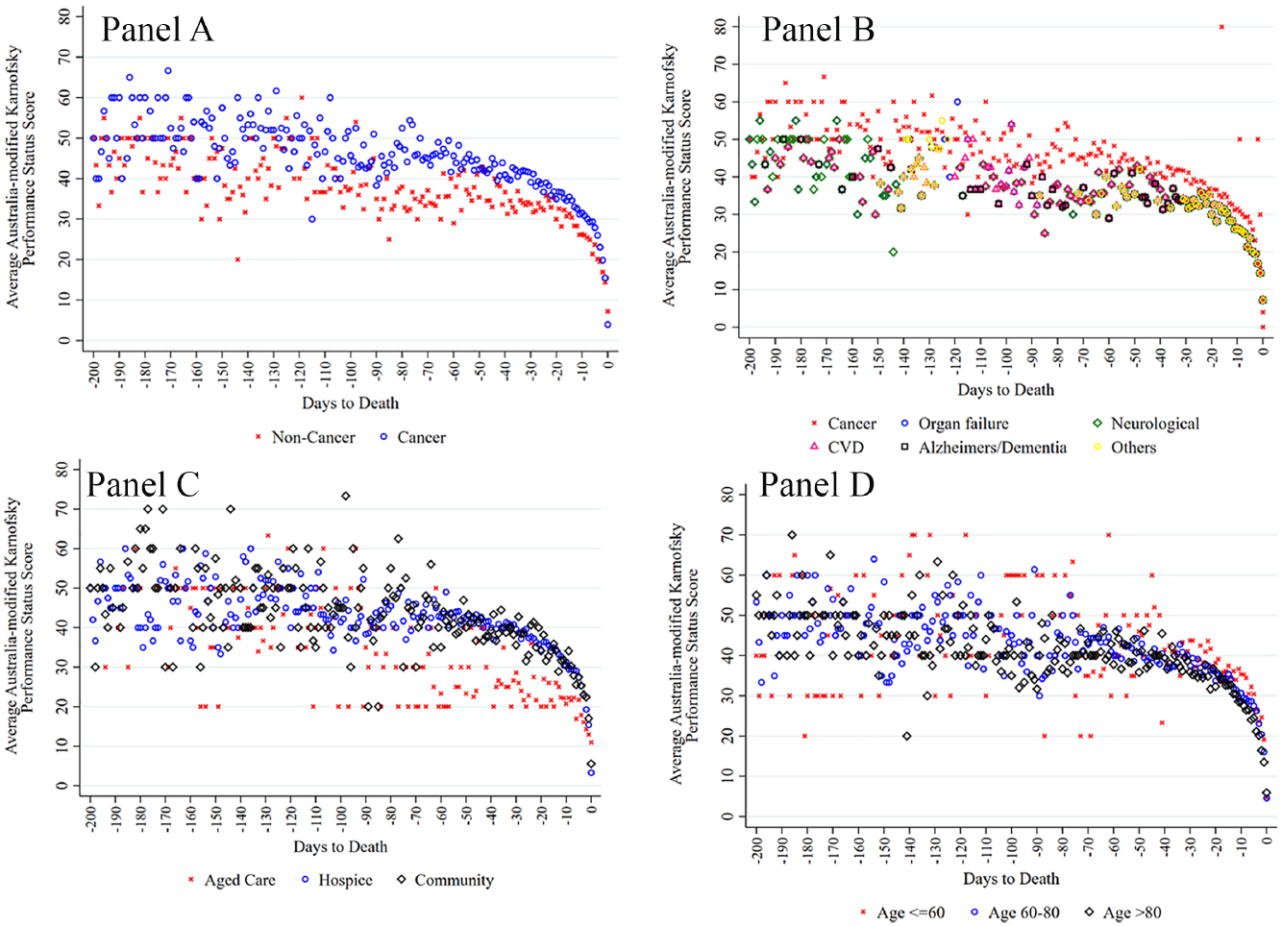
Average Australian-modified Karnofsky Performance Status. Panel A: By cancer. Panel B: By primary diagnosis. Panel C: By location. Panel D: By age group.

Figure 2 reports the accumulated average AKPS scores of the 786 included patients over a 200-day period prior to death. A few interesting patterns emerged from these figures. First, regardless of diagnosis or location there was a tipping point at around 20–30 days prior to death where there was a rapid decline, where the average AKPS score was 30–40 (Panels A–C). Interestingly, for the younger cohort (aged 18–60 years, *n* = 45, 6%), the tipping point hovers between 10 and 20 days before death (Panel D). The average AKPS is slightly lower among those who have non-cancer disease (Panel A) which reflects the sample from aged care homes (Panel C) for patients with dementia or other non-cancer diseases.

**Figure 3.**
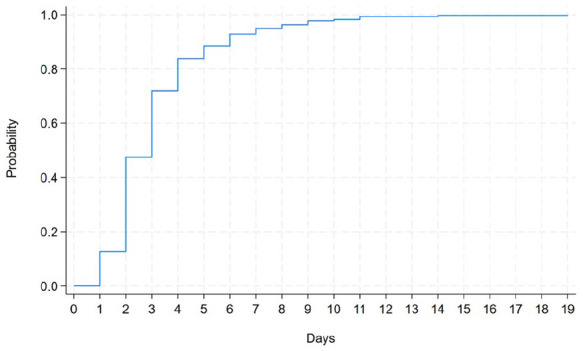
Interval-Censored Cox failure curve after adjusting for age, diagnosis, gender and standard deviation of AKPS score for the last 7 days.

Kruskal-Wallis test results show that patients with or without cancer had similar probability of not surviving (*p* = 0.48). Results were similar for primary diagnosis (*p* = 0.54), location (*p* = 0.41) and age (*p* = 0.24). Figure 3 reports the adjusted Interval-Censored Cox failure curve. Having adjusted for age, cancer, gender, the standard deviation of AKPS for the 7-day period prior to death, the likelihood of death within 2 days is 47%. Most people (84%) will have died within 4 days of being assessed as comatose or barely rousable (Figure 3).

## Discussion

### Predicting time to death

The AKPS score of 10 has been used to predict the time to death and to answer the ‘how long do you think’ question at end of life when the patient is unresponsive and dying. Almost half of the included population died on the second day of being assessed as comatose or barely rousable regardless of location, and most people had died within 4 days. Previous research using the Palliative Performance Scale (PPS) 10% scores, which has been assessed as being interchangeable with the AKPS,^34^ found a median survival time of 2 days for those with a mixed diagnosis (cancer and non-cancer).^7,21,22,35^ The findings of the current study indicated the median was 1 day (mean = 2.1). Although similar, differences in findings may be related to the specificity of our patient cohort. This research specifically aimed to determine the length of time to death once the patient was unresponsive and deemed to have an AKPS of 10 which provides new insights. Other research has only utilised AKPS scores that were submitted at a change of Phase and then retrospectively examined from the date of death.^20^ A recent systematic review using PPS scores reported that there was a wide variation in how the assessment tool was used, and how scores were recorded and reported leading to further uncertainty.^24^

### Diagnosis: Cancer versus non-cancer and time to death

There is limited previous research which include timeframes to death for those with cancer versus non-cancer in the last hours and days of life.^24,36^ As with the results of our study, that research found no difference in timeframes between patients with or without cancer who were comatose or barely rousable.^24,36^ In a more recent study however findings were not conclusive, and further research was recommended.^21^ Although the previous results indicate similar timeframes to death when scoring AKPS 10,^24,36^ patients with chronic non-cancer disease may require full care for a more prolonged period, that is AKPS 20, in comparison to those with a cancer diagnosis. Further research to examine AKPS 20/PPS 20% scores across cancer and non-cancer diagnosis with larger cohorts is recommended^21^ and needed.

### Dementia and time to death

Eighteen percent of the cohort in this study were living and dying with Alzheimer’s or some other form of dementia. Despite increasing research relating to survival timeframes from diagnosis to death for this cohort, no research evidence was found examining time from becoming unresponsive to death. This study indicates that patients with or without dementia have a similar probability of not surviving once they score an AKPS 10. Recent research indicates that those dying with dementia have high levels of suffering and call for development of a dementia specific palliative care pathway.^37–39^

### Gender, age, location and time to death

Similar to other research, gender was not a factor in length of time to death.^21^ The mean age of those with an AKPS of 10 was 81 years, with more than a third of the cohort included in this study being residents of an aged care home. Mean age in previous mixed studies examining survival of those who were comatose or barely rousable was between 70 and 75 years old, however they did not include any residents of an aged care homes, which may account for the slight increase in mean age in this study.^21,23,36^ Most of the patient cohort who were under 60 years of age in this research had a cancer diagnosis. Similarly other studies found that the younger patients who died with an AKPS of 10 predominantly had a cancer diagnosis.^21,24^ As with other studies a non-cancer diagnosis was more dominant among the older cohort of dying patients.^21,24^

### Outliers and time to death

Although most people who were recognised as unresponsive and imminently dying had died within a week, there was a small percentage who had a prolonged terminal phase. The reason for the prolonged AKPS 10 of the youngest of our study cohort may be explained by the pathophysiology and clinical progression of glioblastoma multiforme. Prolonged reduction in consciousness level in this patient cohort is often linked to the medication regimes prescribed to manage symptom burden such as seizures.^40,41^ The clinical notes of the other patients whose terminal phase lasted longer than 7 days were examined but no corresponding reason for their longevity was revealed. Conveying and acknowledging uncertainty is important,^19^ yet having a more confident estimate as presented by this research will extend clinicians confidence in prognostic accuracy, and will help guide and facilitate timely supported conversations.^42^ End-of-life clinical plans should include research evidence, such as findings from this study, to support decision-making and good communication.

### Those who remain alert

One of the implications of 49% of people being unresponsive for longer than 24 h prior to death is that 51% of patients remain alert, as indicated by an AKPS score of 20 or higher in the last 24 h prior to death. The state of alertness of more than half the dying population could suggest that neither family nor clinicians may be aware death may be imminent in the next 24 h. Using the AKPS as a prognostic tool to aid decision-making, further research of AKPS scores is required, and in particular AKPS 20 as a prognostic guide for AKPS 10 and therefore death.

### Strengths

The strength of this study is that it utilises all AKPS scores allocated at each point of care from admission to the specialist palliative care service, all outliers were accounted for and therefore represents a robust account of the last hours and days of life. To provide an accurate representation of the length of time till death for those who were unresponsive, time to death was only assessed across those scoring an AKPS 10 rather than an average across all patients AKPS scores. Both cancer and non-cancer diagnosis were included in this study, and included people across the adult age span where specialist palliative care was provided in a hospice, community and in aged care homes. Other studies provided an AKPS average score,^20^ only focussed on those with cancer,^43^ or only utilised one location,^21^ therefore this study provides valuable new insights.

## Limitations

This study has several limitations that need to be taken into consideration. Firstly, the retrospective nature of the study design means that the study is dependent on the data entered into the clinical database. Every effort was made to ensure missing data was accurately retrieved from clinical notes by two researcher clinicians with a third researcher to ensure agreement, however this study design remains a limitation. Secondly, only those referred to specialist palliative care were included in this dataset. Data from this study, although including three different settings, represents a single specialist palliative care service, with only 10 months of data. These limitations need to be taken into account when considering the results as it may not be generalisable to all dying populations. Thirdly, it is acknowledged that Aboriginal and Torres Strait Islander people were under-represented. Fourthly, allocation of AKPS scores are based on the subjective assessment and decision-making of individual clinicians at the time of care provision. Limitations associated with left-censored data were addressed using an Interval-Censored Cox Proportional Hazards model. Bias secondary to lack of homogeneity in assessments needs to be taken into consideration. Equally the influence of different levels of care and service provision between the locations was not accounted for and is acknowledged as a further limitation. Finally, the study was strongly informed by the recorded primary diagnosis. The influence of comorbidities as confounding variables was not taken into consideration.

## Conclusion

This study provides valuable new knowledge to support clinicians’ confidence when responding to the ‘how long’ question at end-of-life. The findings identify an association between the AKPS 10 score and timeframes to death. These findings could be incorporated into end-of-life clinical care plans to support decision-making and good communication. Multi-centre studies are needed using larger cohorts to replicate and validate these findings. Follow up research to investigate further uses of AKPS scores in predicting length of time to death, and in particular AKPS 20 as a predictor of AKPS 10 and death, is essential.
